# MANAGEMENT OF FLAP DEHISCENCE AFTER LıMBERG PROCEDURE FOR RECURRENT PıLONıDAL DıSEASE BY NEGATıVE PRESSURE WOUND THERAPY (NPWT)

**DOI:** 10.1590/0102-6720201700010021

**Published:** 2017

**Authors:** Sukru TAS, Omer Faruk OZKAN, Muzaffer Muazzez OCAKLI, Emrah ARSLAN, Asli KIRAZ, Muammer KARAAYVAZ

**Affiliations:** 1 Canakkale Onsekiz Mart University, Faculty of Medicine, Department of General Surgery, Canakkale, Turkey;; 2 Canakkale Onsekiz Mart University, Faculty of Medicine, Department of Plastic and Reconstructive Surgery, Canakkale, Turkey;; 3 Canakkale Onsekiz Mart University, Faculty of Medicine, Department of Medical Microbiology, Canakkale, Turkey.

**Keywords:** Surgical dehiscence, Pilonidal cyst, Surgery.

## INTRODUCTION

Sacrococcygeal pilonidal disease is a common inflammatory process affecting young adults. This is mostly seen in sacrococcygeal region. There are multiple factors, which can basically be divided into mainly two, as congenital (such as a result of fusion failure, deeper localized natal cleft) and acquired (such as local infection) factors^1^ ^,^ ^8^. Non-operative and operative strategies are mainly used in management. Local flap use is accepted as the favorite surgical closure method with high success rates, once the lesion is excised. On the other hand, surgical approach occasionally may fail and so several complications are seen such as infection, hemorrhage and flap dehiscence^1^ ^,^ ^4^.

When a complication occurs, a precise wound care is needed to manage the wound properly. Herein we present a case with flap dehiscence and infection following a local flap closure in the management of a recurrence of a pilonidal disease usıng a negative pressure wound therapy (NPWT).

## CASE REPORT

A 66 year old, disabled female was admitted to general surgery clinic with secretions from an orifice in upper edge of flap and abscess formation localized in sacrococcygeal region. It was noted that the patient had a Limberg flap surgery for pilonidal sinus disease one year ago and a hip protesis five years ago. A surgical drainage was planned and performed. In medical treatment, ceftriaxone and metronidazole were administered.

**FIGURE 1:**
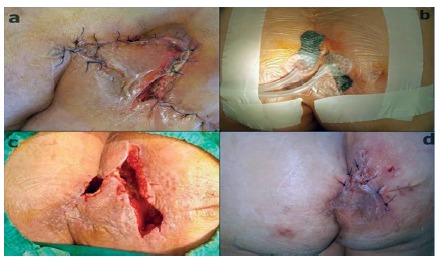
A) Infection and flap dehiscence prior to treatment; B) replacement of NPWT device; C) wound with granulation tissue after NPWT; D) wound sutured

After no infection was seen, the patient underwent a second rhomboid excision and Limberg flap procedure by preparing left side gluteal flap. At postoperative 7^th^ day, an infection reoccured despite antibiotic administration. Then flap dehissenced (Figure 1A). A new debridement was performed and followed by a negatıve pressure wound therapy (NPWT, Confort-Turkey). NPWT was continued for nine days, with a dressing change every 72 h. The pressure was 60 mmHg, and continued with 5 min on and off intervals with instillation of saline (Figure 1B). At the end of the 10^th^ day, the wound was ready to suture with sufficient granulation formation (Figures 1C and 1D).

## DISCUSSION

NPWT is one of the treatment approaches to increase healthy granulation tissue for complex wounds^7^. It is also known that NPWT is an effective therapy decreasing bacterial contamination in wounds^5^. Recently, there are few reports about its successful use in the management of pilonidal sinus disease and recurrent form in addition to surgical treatment^2^ ^,^ ^3^. In the literature, there is no study regarding the use of NPWT for the flap dehiscence.

Infection and dehiscence after flap surgery may lead to removal of flap and a secondary surgical intervention is required to close large sized tissue defects^9^. Prolonged hospital stay, high treatment cost, and late return to work are among disadvantages. When NPWT is used, granulation tissue formation increases with the mechanisms of increased blood flow, and aspiration of infected materials and exudates. NPWT increases blood flow in the applied area and thus works in favor of any flap tissue remaining even though partially lost. This gives an opportunity to use the same flap to close the wound in most cases^6^ ^,^ ^10^. So, on the basis of this case, it can be suggested that the usage of NPWT promotes wound healing and contributes to the flap survival in the presence of infection and flap dehiscence in recurrent pilonidal disease.
